# From the regulatory mechanism of TFEB to its therapeutic implications

**DOI:** 10.1038/s41420-024-01850-6

**Published:** 2024-02-16

**Authors:** Huixia Chen, Siqiao Gong, Hongyong Zhang, Yongming Chen, Yonghan Liu, Junfeng Hao, Huafeng Liu, Xiaoyu Li

**Affiliations:** 1 Institute of Nephrology, and Guangdong Provincial Key Laboratory of Autophagy and Major Chronic Non-Communicable Diseases, Affiliated Hospital of Guangdong Medical University, Zhanjiang, 524001 China; 2 Zhanjiang Institute of Clinical Medicine, Central People’s Hospital of Zhanjiang, Guangdong Medical University Zhan-jiang Central Hospital, Zhanjiang, 524001 China

**Keywords:** Autophagy, Post-translational modifications

## Abstract

Transcription factor EB (TFEB), known as a major transcriptional regulator of the autophagy-lysosomal pathway, regulates target gene expression by binding to coordinated lysosomal expression and regulation (CLEAR) elements. TFEB are regulated by multiple links, such as transcriptional regulation, post-transcriptional regulation, translational-level regulation, post-translational modification (PTM), and nuclear competitive regulation. Targeted regulation of TFEB has been victoriously used as a treatment strategy in several disease models such as ischemic injury, lysosomal storage disorders (LSDs), cancer, metabolic disorders, neurodegenerative diseases, and inflammation. In this review, we aimed to elucidate the regulatory mechanism of TFEB and its applications in several disease models by targeting the regulation of TFEB as a treatment strategy.

## Facts

1. The regulation of TFEB by phosphorylation is contradictory. Phosphorylation negatively regulates TFEB by controlling its subcellular localization; however, positively regulates TFEB by enhancing its transcriptional activity.
2. Each regulatory mechanism of TFEB is not universal to most cells.
3. TFEB agonists have been successfully used in several disease models, and the first TFEB inhibitor is available.

## Open questions

1. Is the regulatory mechanism of TFEB validated under in vitro physiological conditions still applicable to some pathological situations in vivo?
2. Is the long-term application of TFEB agonists as a disease treatment strategy safe?
3. Is the use of TFEB inhibitors as a therapeutic strategy for some cancers can achieve clinical therapeutic effects?

## Introduction

TFEB, a member of the microphthalmia (MiT/TFE) family of leucine zipper transcription factors, has been known as a master regulator for transcription of genes participated in lysosome biogenesis and autophagy [1, 2]. Except for TFEB, MiT/TFE family members include transcription factor EC (TFEC), microphthalmia-associated transcription factors (MITF), and transcription factor E3 (TFE3). TFEB forms homo-or heterodimers with itself or other MiT/TFE family members and binds to CLEAR, thereby activating the expression of autophagy-lysosomal genes [3]. In addition to autophagy-lysosome biogenesis, TFEB is involved in metabolic processes and cellular energy homeostasis in response to internal and external stresses [4].

**Fig. 1:**
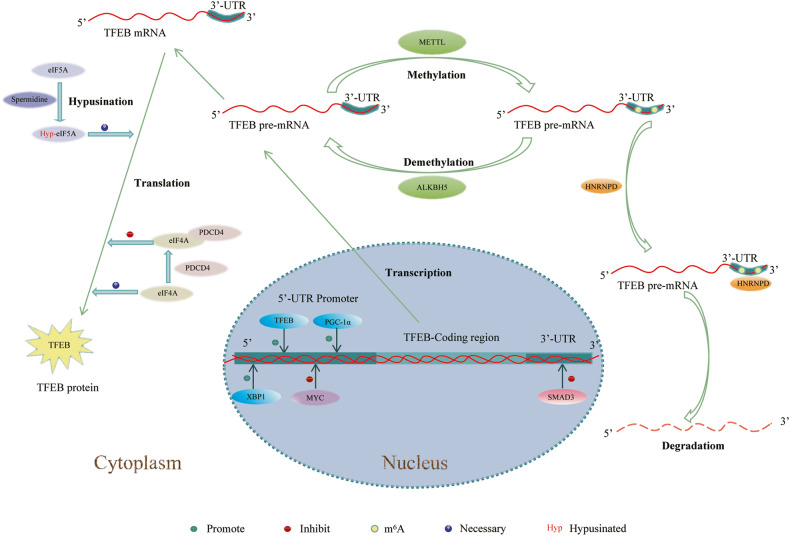
A graphic of the regulatory mechanism of TFEB from transcriptional level, post-transcriptional level, and translational level. TFEB, PGC-1 α and XBP1 bind to the promoters of TFEB to promote its expression. MYC binds to the repressor element in the TFEB promoter region to inhibit its expression. SMAD3 binds to the 3’-UTR of TFEB to inhibit its transcription. METTL3 identifies and methylates two m^6^A consensus sequence in the TFEB pre-mRNA 3’-UTR. Methylated TFEB pre-mRNA bind with HNRNPD, resulting in degradation of the TFEB pre-mRNA. Meanwhile, methylated TFEB pre-mRNA can be demethylated by ALKBH5. eIF5A is hypusinated by spermidine, promoting the synthesis of TFEB because the triproline motif on the TFEB protein requires hypusinated eIF5A for efficient synthesis. PDCD4 interacts with eIF4A inhibiting TFEB protein generation.

The expression and activity are regulated by multiple mechanisms, including transcriptional [5, 6], post-transcriptional [7], translational [8, 9], PTM [10], and nuclear competitive regulation [11, 12]. The transcriptional, post-transcriptional, and translational levels regulate expression of TFEB [6, 7, 9] (Fig. 1). PTM and nuclear competitive regulation mainly regulate activity of TFEB [3, 11].

As TFEB plays a crucial role in the regulation of various cellular processes, its dysregulation is associated with a host of human diseases, such as lysosomal storage disorders [13], cancers [14], metabolic disorders [15, 16], inflammation [17–19] and neurodegenerative diseases [20]. In this review, we elucidate the regulatory mechanisms of TFEB and the latest research results on the application of these mechanisms to disease treatment.

## The regulatory mechanisms of TFEB

### Transcriptional regulation

Transcriptional regulation is an important process in eukaryotic gene expression. Multiple factors can regulate the transcription of TFEB [6, 21, 22]. As a transcription factor, TFEB also can promote its own expression [5]. Settembre et al. found that in starved mouse livers, TFEB regulates its own expression by directly binding to the CLEAR in its promoter [5]. Peroxisome proliferator-activated receptor γ coactivator 1α (PGC-1α), a transcription factor [23], was found to promote the expression of TFEB by directly ligating promoter of *Tfeb* [6]. PGC-1α was found to promote huntingtin turnover and the clearance of protein aggregates by activating *Tfeb* in Huntington’s disease (HD) transgenic mice [6]. X-box binding protein 1 (XBP1), a member of the CEB/P family of transcription factors, binds to UPR elements (UPRE) on their target genes. Zhang et al. found that, in the mouse liver, XBP1 promotes the expression of *Tfeb* by binding to UPRE on its promoter. Their data also suggest that XBP1 activates hepatic autophagy by upregulating the expression of *Tfeb* [21]. MYC has been reported to act as a transcriptional repressor to inhibit the expression of TFEB. Suzuki et al. found that in human retinal pigment epithelial-1 cells, MYC repressor complexes bind to the MYC response element on the TFEB promoter to inhibit its expression [22].

As signal transducers for the transforming growth factor-β (TGF-β), smads have also been reported to regulate TFEB [24, 25]. Chen et al. found that hyperactivated SMAD3 triggers lysosomal depletion by inhibiting the expression of TFEB in human proximal tubular epithelial cell line (HK-2) under diabetic conditions [25]. They further found that SMAD3 bind directly to the 3’-untranslated region (3’-UTR) of *TFEB* and inhibit its transcription [25].

### N^6^-Methyladenosine (m^6^A) Modification

M^6^A modification, one of the most common, abundant, and conserved post-transcriptional modifications [26], is a dynamic and reversible process that is induced by methyltransferases, reversed by demethylases, and mediated by m^6^A-binding proteins [27]. Highly expressed methyltransferase-like 3 (METTL3) negatively regulates autophagy flux by decreasing the expression of TFEB in hypoxia/reoxygenation-treated cardiomyocytes [7]. Mechanistically, METTL3 identify two m^6^A consensus sequence 5’-RRACU-3’ in the TFEB 3’-UTR and methylates the adenosine, which promotes the association of the RNA-binding protein heterogeneous nuclear ribonucleoprotein D (HNRNPD) with TFEB pre-mRNA, leading to rapid degradation of the TFEB pre-mRNA and reduces TFEB protein production [7]. Supplementation with the RNA demethylase *AlkB* family protein 5 (ALKBH5) can reverse this effect because ALKBH5 demethylates TFEB to promote its expression [7]. Moreover, TFEB promotes ALKBH5 transcriptional activation, thereby establishing a positive feedback axis [7].

### Translational regulation

Translational regulation also plays an important role in the regulation of eukaryotic gene expression. To date, only two molecules have been reported to regulate the synthesis of TFEB proteins by affecting translation initiation factors [8, 9]. Spermidine, an endogenous polyamine metabolite, was found to involved in hypusination, a post-translational modification of eukaryotic initiation factor 5 A (eIF5A) [28]. Zhang et al. found that TFEB-driven autophagy is induced in B cells from old mice treated with spermidine for 6 weeks [8]. They further found that hypusinated eIF5A by spermidine directly facilitated the synthesis of TFEB because the triproline motif on the TFEB protein requires hypusinated eIF5A for efficient synthesis [8]. Programmed cell death 4 (PDCD4), a tumor suppressor, is a binding protein of eukaryotic initiation factor 4 A (eIF4A), which inhibits translation [29]. The expression of TFEB was elevated, and lysosome amounts were increased by approximately two times in *Pdcd4*-deficient mouse embryonic fibroblasts (MEFs). The knockdown of TFEB by small interfering RNA reversed this increase in lysosomal amounts [9]. Mechanistically, PDCD4 suppresses the structured 5′UTR mRNA of TFEB via binding to eIF4A, thereby inhibiting the initiation translation of TFEB and TFEB protein generation [9].

### PTM

The activity and subcellular localization of TFEB are mainly regulated by PTM, including phosphorylation [3], acetylation [30], ubiquitination [31], Poly-ADP-ribosylation (PARsylation) [32], SUMOylation [33], glucosylation [34], oxidation [35, 36] and sulfhydration [37]. The PTMs of TFEB that have been reported to date are summarized in Table 1.

**Table 1:** Post-translational modifications of TFEB that have been reported to date (S: serine, K: lysine, T: threonine, C: Cysteine).

Site	PTM	Kinase	Effects of site PTM on TFEB	References (PMID)
S211	Phosphorylation	mTORC1	Cytoplasmic retention	28055300
		p38 MAPK		34930303
	Dephosphorylation	Calcineurin	Nucleus translocation	26043755
S142	Phosphorylation	mTORC1	Cytoplasmic retention	22343943
			Nuclear export	30120233
		ERK2	Cytoplasmic retention	21617040
			Nuclear export	29992949
		CDK4/6	Nuclear export	32662822
	Dephosphorylation	Calcineurin	Nucleus translocation	26043755
S138	Phosphorylation	mTORC1	Nuclear export	30120233
		GSK3β	Lysosomal localization	27617930
			Nuclear export	29992949
	Glucosylation	–	Blocking TFEB nuclear export	32622269
S122	Phosphorylation	mTORC1	Cytoplasmic retention	21617040
	Dephosphorylation	PP2A	Nucleus translocation	29945972
S467	Phosphorylation	Akt	Cytoplasmic retention	28165011
		AMPK	Enhanced transcriptional activity	33734022
S466		AMPK	Enhanced transcriptional activity	33734022
		PKCβ	Promoting its stability	23599343
S469		AMPK	Enhanced transcriptional activity	33734022
S461/S462 and S468		PKCβ	Promoting its stability	23599343
S3		MAP4K3	Cytoplasmic retention	29507340
S401		p38 MAPK	Nucleus translocation	36507874
S134		GSK3β	Lysosomal localization	27617930
S109, and S114	Dephosphorylation	PP2A	Nucleus translocation	29945972
K116	Acetylation	ACAT1	Hindering TFEB binding to the DNA	30059277
	Deacetylation	SIRT1	Enhancing transcriptional activity	27209302
K91, K103 and K430	Acetylation	ACAT1	Promoting nuclear translocation and binding to DNA	30059277
K274 and K279		GCN5	Hindering TFEB binding to the DNA	31750630
-	Ubiquitination	STUB1	Enhancing activity	28754656
-	PARsylation	TNKS1	Nucleus translocation	33753903
K316	SUMOylation	–	Attenuating its transcriptional activity	37950772
S195, S196, S203, T201and T208	Glucosylation	–	Promoting nuclear translocation	32622269
C212	Oxidation	–	Inhibiting phosphorylation of S211 by mTORC1 and producing oligomers to enhance its activity	33314217
		–	Inhibiting the binding of TFEB to Rag GTPases	31826695
	S-sulfhydration	CTH-H_2_S	Promoting nuclear translocation	35090378

#### Phosphorylation

According to large-scale phosphoproteomics studies, multiple serine residues in TFEB are phosphorylated [38, 39]. The regulation of TFEB by phosphorylation is contradictory. On one hand phosphorylation mainly negatively regulates TFEB by controlling its subcellular localization [40, 41]. Under basal conditions, phosphorylated TFEB is primarily inactive in the cytoplasm [40]. Nevertheless, under cellular stress conditions, such as starvation, DNA damage, or oxidative stress, dephosphorylated TFEB translocates to the nucleus to bind to the CLEAR element, in turn promoting the expression of its target genes [42, 43]. TFEB can also be phosphorylated in the nucleus, promoting shuttling from the nucleus to the cytoplasm [44]. On the other hand, the phosphorylation of some serine sites in TFEB does not affect its subcellular localization, but rather enhances its transcriptional activity [45, 46]. Different serine sites of TFEB phosphorylated by the same kinase produced different results [46, 47]. Moreover, phosphorylation of the same site by different kinases can also have different effects [45, 48].

##### S211

To investigate the effect of serine phosphorylation on the subcellular localization of TFEB, researchers usually mutated TFEB serine (S) to alanine (A) to mimic the unphosphorylated state and to aspartate (D) to mimic the phosphorylated state [49]. However, whether the aspartate substitution affects TFEB localization by mimicking phosphorylation remains unclear [49].

**Fig. 2:**
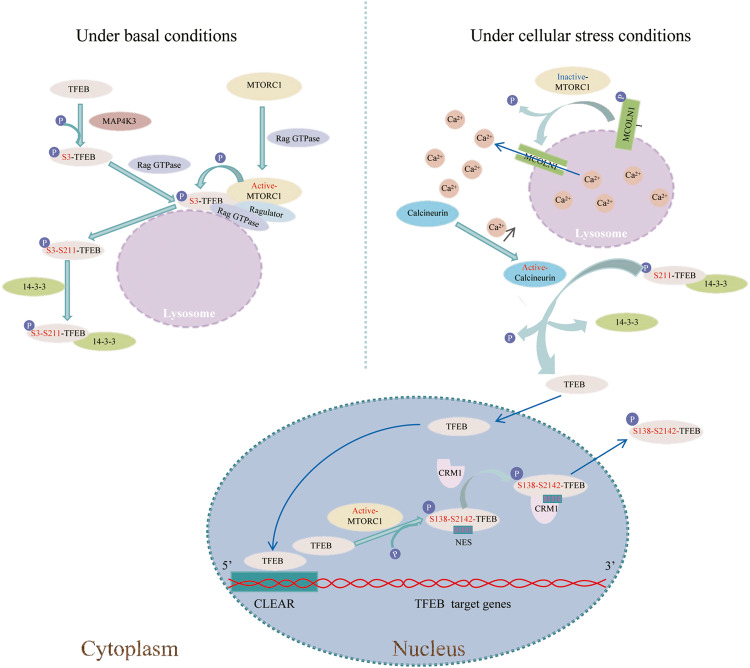
A proposed graphic of TFEB S211 phosphorylation-mediated cytoplasmic localization and nuclear export via mTORC1 and dephosphorylation-mediated nuclear translocation via Calcineurin. Under basal conditions, Ragulator interacts with the Rag GTPases and anchors Rag GTPases to the lysosomal surface. MAP4K3 phosphorylates S3 in TFEB, which enables the Rag GTPases to recruit TFEB to lysosomal surface. Meanwhile, mTORC1 moves to lysosomal membranes, where the Rag proteins reside, interacts with Rag GTPases, and is activated by it. Subsequently, activated mTORC1 phosphorylates S211 in TFEB, which can lead to TFEB interact with 14-3-3 and remain in the cytoplasm. Under cellular stress conditions, Rag GTPases are inactivated, resulting in an inactivation of mTORC1 and then mTORC1 dissociate from the lysosomal surface. Meanwhile, inactivated mTORC1 is no longer capable of phosphorylating MCOLN1. Activated MCOLN1 triggers Ca^2+^ release from lysosomes and elevation of cytosolic Ca^2+^ activates Calcineurin, which dephosphorylates TFEB at S211, thereby contributing to its nuclear translocation. TFEB bind to the CLEAR element to promote the expression of its target genes. Activated mTORC1 phosphorylates TFEB at S138 and S142 in the nucleus, which may enable the NES exposure. CRM 1 recognizes the NES of TFEB and interact with TFEB leading to its nuclear export.

The mechanistic target of rapamycin complex 1 (mTORC1), an atypical serine/threonine kinase, controls the balance between anabolism and catabolism and responds to various signals, including nutrients [50]. mTORC1-mediated phosphorylation of TFEB is a complex process (Figure 2). Ragulator, a pentameric protein complex, interacts with the Rag GTPases and recruits them to the lysosomes [51]. Rag GTPases heterodimers consisted of Rag A or B linked to Rag C or D [52]. In HeLa cells, mTORC1 moves to lysosomal membranes, where the Rag proteins reside, bind to Rag GTPases, and is activated by them in response to amino acid stimulation [51]. Martina et al. found that in HeLa cells, TFEB interacts with active Rags and associates with lysosomes in response to amino acid stimulation [53]. They further found that the first 30 amino acids of TFEB were required for interaction with Rag and lysosomal distribution [53], which is consistent with the results of Hsu et al. [54]. Activated mTORC1 phosphorylates TFEB at S211 on lysosomal membranes, which is required for the binding of TFEB to 14-3-3 protein to retain TFEB in the cytosol [40]. It is presumed that the binding masks the nuclear localization sequence (NLS), thereby inhibiting TFEB nuclear translocation [55]. Besides, S211 of TFEB can also be phosphorylated by p38 MAPK [47], a member of the Mitogen-activated protein kinases (MAPKs) family [56]. Chen et al. found that p38 MAPK inhibitor SB203580 activates TFEB-mediated autophagy in α-synuclein A53T transgenic mice brain [47]. They further found that p38 MAPK suppressed TFEB function by phosphorylating TFEB at S211, thus inhibiting TFEB nuclear translocation [47].

Calcineurin, a calmodulin-dependent serine/threonine protein diphosphatase [57], can dephosphorylate S211 of TFEB [58]. During starvation, Rag GTPases are inactivated, resulting in the inactivation of mTORC1 and then mTORC1 dissociate from the lysosomal surface [49]. Inactivated mTORC1 is no longer capable of phosphorylating mucolipin 1 (MCOLN1, also called TRPML1) [59, 60], a calcium channel present on the lysosomal membrane; and thus, MCOLN1 is activated [59, 61]. Activated MCOLN1 triggers Ca^2+^ release from lysosomes and the elevation of cytosolic Ca^2+^ activates calcineurin, which dephosphorylates TFEB at S211. Therefore, 14-3-3 and TFEB were separated, contributing to its nuclear translocation [58].

##### S142 and S138

Settembre et al. revealed that S142 is a key residue for the phosphorylation of TFEB by mTOR using an mTOR kinase assay [62]. Both S142A and S211A mutations resulted in nuclear localization of TFEB [62]. Similar to S211, phosphorylation of S142 by mTORC1 enables the localization of TFEB to the cytoplasm. S142A has no effect on the formation of the TFEB/14-3-3 complex [40], which is inconsistent with the results of Li et al. [63]. This implies that TFEB S142 phosphorylation is localized to the cytoplasm, independent of binding to the 14-3-3 protein. On the contrary, the results of Li et al. showed that the S142A mutation reduced the phosphorylation of the 14-3-3-binding motif (S211) [63]. Another study showed that in HeLa cells, extracellular regulated protein kinase 2 (ERK2), phosphorylates TFEB at S142, allowing its cytosolic retention [64]. Besides, Calcineurin can dephosphorylated S142 of TFEB, resulting in its nuclear translocation [58].

Glycogen synthase kinase 3β (GSK3β) was found to phosphorylates TFEB at S134 and S138 in HeLa cells, which is required for the localization of TFEB on lysosomes [63]. This probably determines the availability of TFEB for phosphorylation by mTORC1 on lysosomes [40, 63]. Moreover, it needs to be further confirmed whether TFEB phosphorylation by GSK3β mediated its lysosomal localization requires TFEB interaction with Rag.

Phosphorylation of TFEB S142 and S138, affects not only its nuclear translocation [58, 63, 64] but also its nuclear export [41, 44, 65]. Chromosomal maintenance 1 (CRM1) is a receptor for leucine-rich nuclear export signals [66]. Starvation induces nuclear translocation through TFEB dephosphorylation [60]. TFEB is rapidly redistributed from nucleus to the cytosol upon nutrient replenishment, which requires CRM1 and phosphorylation S142 and S138 of TFEB [41, 44, 65]. Treatment of HeLa cells with leptomycin B, a CRM1 inhibitor, severely impairs nuclear export of TFEB upon nutrient refeeding [41, 65]. There is a nuclear export signal (NES) in the N-terminal portion of the TFEB protein, and the mutants of three different hydrophobic residues within the presumed NES, namely I149, L147, and M144, completely impairs the cytosolic relocalization of TFEB following refeeding [41, 65]. In addition, TFEB S142A and S138A mutants showed highly impaired export kinetics [41, 65]. These results imply that the phosphorylation of S142 and S138 of TFEB allows its nuclear export, which may be achieved by the recognition of NES on the TFEB protein by CRM1 [41]. The nuclear pool of mTOCR1 may be responsible for TFEB phosphorylation and induction of nuclear export [41]. Another research shows that phosphorylation S142 by mTOCR1 or ERK stimulates phosphorylation S138 by GSK3β and that the dual phosphorylation event of S142 and S138 is required for nuclear export [65].

Moreover, the phosphorylation of TFEB at S142 by cyclin-dependent kinase 4/6 (CDK4/6) in the nucleus is CRM1-dependent and promotes nuclear export [44]. Two commercial compounds, PD0332991 (palbociclib) and LY2835219 (abemaciclib), which specifically inhibit CDK4/6, induce TFEB-dependent lysosomal biogenesis by inhibiting the nuclear export of TFEB in HeLa cells [44].

##### S122

The S122A single mutant was primarily localized in the cytosol, similar to wild-type TFEB [49], which means the phosphorylation of S122 is not important for the localization of TFEB to the cytoplasm. However, despite the dephosphorylation of S211, the S122D phosphomimetic mutation of TFEB largely blocked the effect of Torin1 (an mTORC1 inhibitor) on nuclear localization of TFEB [49], indicating that dephosphorylation of S122 is necessary for TFEB nuclear localization upon treatment with Torin1. Taken together, these results suggest that TFEB is regulated by mTORC1 in a multistep process that involves at least two residues, S122 and S211 [49].

Chen et al. in HEK293T cells and mouse primary hepatocytes confirmed that protein phosphatase 2 (PP2A), a serine/threonine phosphatase, interacts with TFEB and dephosphorylate it [67]. However, they did not determine the serine residue of TFEB that was dephosphorylated by PP2A [67]. Martina et al. performed in vitro phosphatase assays in HeLa cells and found that PP2A dephosphorylated TFEB at S109, S114, and S122 to promote it nucleus translocation [68].

##### S466, S467, and S469

AMP-activated protein kinase (AMPK), a serine/threonine kinase, directly phosphorylates TFEB to promote its transcriptional activity [45]. Upon the loss of folliculin (FLCN), a Rag C/D activator follicle protein, TFEB is permanently localized to the nucleus because mTORC1 cannot phosphorylate TFEB [69]. Upon deletion of AMPK in *Flcn* KO MEFs, the expression of the TFEB target genes decreased, although its nuclear localization was not reduced [45]. AMPK was found to phosphorylate TFEB on the highly conserved serine clusters S466, S467, and S469 in HEK293T cells [45]. Mutations of S466A, S467A, and S469A lead to elimination of TFEB activation upon AMPK activation or mTORC1 inhibition [45]. These results suggested that AMPK-mediated phosphorylation of S466, S467, and S469 on TFEB is a conformational change that renders TFEB transcriptionally active [45]. In addition, mutations of S466A, S467A, and S469A did not influence its nuclear localization [45], contradicting the results reported by Parmieri et al. [48]. Palmieri et al. using an in vitro Akt kinase assay found that Akt phosphorylates TFEB at S467, promoting its cytoplasmic retention [48]. The mutation of S467A showed diminished co-localization and interaction with 14-3-3 and increased nuclear localization of TFEB in HeLa cells [48]. AMPK and Akt may phosphorylate the C-terminal region in a divergent hierarchy, resulting in divergent activities. In addition, PKC β was found to phosphorylate TFEB at S461 and/or S462, S466, and S468 by in vitro kinase assays [70]. Ferron et al. found that PKC β phosphorylate S461 and/or S462, S466, and S468 of TFEB, which can increase its stability resulting in promoting lysosomal biogenesis in osteoclasts [70].

##### Other serine site

Hsu et al. found that in HEK293A cells, the S3A mutation of TFEB abrogated its binding to Rag GTPases, mTORC1, and the Ragulator complex [54]. They further found that Mitogen-Activating Protein Kinase Kinase Kinase Kinase-3 (MAP4K3) interacted with TFEB and phosphorylated it at S3 [54]. Their results indicated that TFEB S3 phosphorylation by MAP4K3 occurs before and is necessary for TFEB S211 phosphorylation by mTORC1 [54].

p38 MAPK phosphorylates TFEB not only at S211 promoting its cytoplasmic retention [47], but also at S401, leading to its nuclear translocation [46]. THP1, a human leukemia monocytic cell line, has been extensively used to characterize monocyte and macrophage activation and differentiation [46]. THP1 monocytes can differentiate into M0 macrophages upon incubation with phorbol 12-myristate 13-acetate (PMA). During incubation, rapid p38 MAPK activation, increased TFEB-S401 phosphorylation, and rapid nuclear translocation of TFEB were observed [46]. They further showed that p38 MAPK phosphorylated TFEB at S401 to promote its nucleus translocation, which was independent of S211 phosphorylation. The mutation of S401A exhibited decreased nuclear accumulation of TFEB during incubation [46]. However, this phenomenon was not observed in differentiated THP1 cells treated with PMA [46]. These results suggested that the role of S401 phosphorylation in promoting TFEB nuclear accumulation was highly specific to monocytes during the early stages of PMA-induced differentiation [46].

#### Acetylation

Acetylation, a common post-translational modification of proteins, is an important means by which organisms adapt to pathophysiological conditions [71]. Acetylation also appeared to dually and inversely regulate TFEB activity, similar to phosphorylation [72–75]. At present, two enzymes have been found that can acetylate TFEB, including general control non‐repressed protein 5 (GCN5) [72] and acetyl-CoA acetyltransferase 1 (ACAT1) [30, 72], and two enzymes have been found that can deacetylate TFEB, including sirtuin deacetylase 1 (SIRT1) [74, 75] and histone deacetylases (HDACs) [30] .

The accumulation of acetylated TFEB in the nucleus and enhanced transcriptional activity of TFEB were observed in HEKT293T cells treated with suberoylanilide hydroxamic acid (SAHA), an HDACs inhibitor [30]. SAHA treatment enhanced the interaction between ACAT1 and TFEB and reduced the interaction between HDAC2 and TFEB [30]. In addition, mutations in the four acetylated lysine sites of TFEB, K91, K103, K116, and K430, significantly impair TFEB activity [30]. These results suggest that acetylation of TFEB at K91, K103, K116, and K430 by ACAT1 make TFEB has a much higher capability for nuclear translocation and DNA binding [30].

In contrast, other studies have suggested that deacetylation of TFEB by SIRT1 promotes its transcriptional activity to upregulate lysosomal biogenesis [73–75]. SIRT1 is a family of NAD-dependent protein deacetylases that contributes to the deacetylation of a variety of mammalian transcription factors in the nucleus [76]. Deacetylation of TFEB at K116 by SIRT1 enhances the activity of TFEB, which accelerates fAβ degradation in microglia by promoting lysosomal biogenesis [74]. Another study showed that the deacetylation of TFEB by SIRT1 promotes its nuclear translocation, which in turn promotes autophagy [73]. In animal cells and Drosophila, GCN5, a histone acetyltransferase, inhibits autophagosome and lysosome biogenesis by targeting TFEB [72]. Mechanistically, the acetylation of TFEB at K274 and K279 by GCN5 interferes with TFEB dimerization, thereby hindering its binding to DNA, which does not affect its nuclear translocation [72]. In summary, acetylation of TFEB by GCN5 decreases its transcriptional activity [72], whereas deacetylation of TFEB by SIRT1 promotes its nuclear translocation and transcriptional activity [73, 74].

#### Ubiquitination

Ubiquitination, performed by Ubiquitinating enzymes (UBEs), can target substrates for degradation through either the ubiquitin-proteasome or the autophagy–lysosome system [77]. STIP1 homology and U‐Box containing protein 1 (STUB1), as a chaperone-dependent E3 ubiquitin ligase, a UBEs, plays a role in regulating TFEB activity. STUB1 preferentially binds to phosphorylated TFEB and degrades it via the ubiquitin-proteasome pathway [31]. Accumulation of phosphorylated TFEB and inhibition of autophagy were observed in STUB1–deficient MEFs cells, which could be rescued by constitutively active TFEB mutants [31]. These results indicated that targeting phosphorylated TFEB for degradation is an important mechanism for enhancing TFEB activity. Notably, targeting of the phosphorylated serine residue of TFEB for ubiquitination and degradation requires further investigation.

#### PARsylation

PARsylation is a reversible PTM performed by poly (ADP-ribose) polymerases (PARPs) that mediate the covalent addition of one or more ADP-riboses to acceptor proteins [78]. Tankyrases are a group of PARPs with two isoforms: tankyrase1 (TNKS1) and tankyrase2 (TNKS2) [79]. Kim et al. found that the nuclear localization of TFEB was increased in HeLa cells overexpressing TNKS1 [32]. They further found that TNKS1 interacts with and parsylates TFEB, promoting its nuclear translocation [32].

#### SUMOylation

SUMOylation, a PTM, is performed by small ubiquitin-like modifier (SUMO), which covalently and reversibly bind to the target proteins and subsequently alter their functions [80]. Miller et al. found that TFEB was sumoylated at K316 in COS-7 cells, but the effect of SUMOylation on the subcellular localization and activity of TFEB is not defined [81]. Wang et al. also verified in 293 T cells that TFEB was sumoylated at K316, which attenuate its transcriptional activity [33], Their results further indicated that TFEB SUMOylation inhibits TFEB binding to facilitates chromatin transcription (FACT) complex, which is a heterodimeric histone chaperone composed of SPT16 homolog, facilitates chromatin remodeling subunit and structure specifc recognition protein 1 [33]. The binding of FACT complex and TFEB can promote the transcriptional activity of TFEB, but the molecular mechanism is not fully understood [82].

#### Glucosylation

Beck et al. found that TFEB was glucosylated by Legionella effector, SetA in Legionella pneumophila, causing nuclear enrichment of TFEB [34]. SetA could glucosylates TFEB at multiple Ser/Thr sites, including S138, S195, S196, T201, S203 and T208, which adjacent to S211 of TFEB except S138. Glucosylation of the S138 blocks TFEB nuclear export and glucosylation of the other Ser/Thr sites disrupts the binding between TFEB and 14-3-3 to promote nuclear translocation of TFEB [34]. Whether the PTM is involved in the regulation of TFEB in spinal animals is not known.

#### Oxidation

Martina et al. found that in HeLa cells and ARPE-19 cells, oxidation, a PTM, was also involved in the regulation of TFEB in response to stress [36]. Only one cysteine residue, C212, exists in the entire sequence of TFEB, which is oxidized to form a ROS-dependent disulfide bond [36]. Cysteine oxidation of TFEB at C212 inhibits phosphorylation of S211 by mTORC1 and produces oligomers with increasing transcriptional activity [35]. Wang et al. also showed that C212 on TFEB is prone to oxidation in HEK293 cells under oxidative stress [35]. Different from the results of Martina et al. [36], their results suggest that oxidation of C212 inhibits the binding of TFEB to Rag GTPases, resulting in diminishing its lysosomal localization and its promoting nuclear translocation [35]. It is unclear about the disagreement between these results, probably due to different experimental conditions.

#### S-sulfhydration

Besides oxidation [35, 36], C212 of TFEB can also undergo S-sulfhydration [37]. which is the chemical modification of cysteine residues by hydrogen sulfide (H_2_S) [83]. C212 of TFEB is sulfhydrated by cystathionine gamma-lyase (CTH)-H_2_S in vascular smooth muscle cells, causing nuclear translocation and enhanced activity of TFEB [37].

### Competitive regulation

Apart from the above regulatory mechanisms, there is a particular mechanism that participates in TFEB regulation called competitive regulation [11]. It does not change TFEB translocation from the cytoplasm to the nucleus but regulates TFEB activity by competing for the CLEAR motif in the nucleus [11, 12]. The inhibitory effect of MYC on TFEB can be achieved by not only binding to its repressor element [22], but also competing with TFEB for binding to the CLEAR element [11]. Overexpressed c-MYC represses the expression of autophagy-lysosomal genes in HeLa cells because c-MYC repressor complexes compete with TFEB for binding to the CLEAR on the promoter of autophagy-lysosomal genes [11]. A recent study has demonstrated the same phenomenon. Mir-30b-5p, is a small RNA known to post-transcriptionally regulate a variety of genes in the cytoplasm. It competes with TFEB to bind to the CLEAR, which inhibits the transcription of TFEB-dependent downstream genes and further inhibits autophagic flux and lysosomal biogenesis [12].

## TFEB in diseases

Originally regarded as an oncogene, TFEB is nowadays widely known as a regulator of multiple processes such as autophagy-lysosomal biogenesis, cellular energy homeostasis, stress response, and metabolism [4]. As TFEB plays a crucial role in the regulation of multiple cellular processes, its dysregulation is related to a host of human diseases. In addition to the finding that TFEB plays a pathogenic role in several cancers [14, 43, 84], TFEB plays a protective role in most diseases, such as LSDs [13, 85], neurodegenerative diseases [6, 86, 87], ischemic injury [88, 89], metabolic disorders [15, 90], and inflammation [17–19]. Disease models that successfully target TFEB as a therapeutic strategy are summarized in Table 2.

**Table 2:** Disease models that successfully target TFEB as a therapeutic strategy.

Targeting regulatory mechanisms of TFEB	Disease	Characteristics of disease	The disease models	Methods of targeting TFEB	The improved phenotype	References (PMID)
Gene therapy	Pompe disease	An LSD and severe metabolic myopathy caused by the deficiency of acid alpha-glucosidase (GAA)	GAA knockdown mice	Intramuscular injection of AAV-TFEB	Increasing clearance of glycogen stores and amelioration of muscle pathology	23606558
	Asthma	The critical drivers of asthma pathogenesis are NLRP3-driven inflammatory responses by circulating and lung-resident monocytes.	A severe asthma (SA) mouse model	Myeloid-specific TFEB-overexpressing mice	Improvement in NLRP3-driven pulmonary inflammation, and SA phenotype.	35038351
	Nephropathic cystinosis	A rare autosomal recessive lysosomal storage disease characterized by accumulation of cystine	Immortalized proximal tubular epithelial cells derived from the urine of a cystinotic patient	Transduction with TFEB-GFP lentiviral vectors.	Reduction of cystine levels within 24 hours in cystinotic cells	26994576
	Permanent cerebral ischemia	Brain ischemic injury	Permanent middle cerebral artery occlusion- operated rats	Transduction with Syn1 promoter-driven-TFEB-AAV particles into the cortex of ischemic stroke rats	Decrease of brain infarct volume	30304977
	Pancreatitis	Increase of serum amylase and lipase activities, pancreatic edema, infiltration of inflammatory cells	A chronic plus acute alcohol binge (referred to as Gao-binge) mouse model	Injection of adenovirus-TFEB (Ad-TFEB)	The decreased levels of serum amylase and lipase as well as decreased pancreatic edema and ZG accumulation	31987928
Transcription level	HD	An autosomal dominant neurodegenerative disorder caused by CAG/polyglutamine repeat expansions in the huntingtin gene	HD transgenic mice	Induction of PGC-1α expression by the transgenic technique (PGC-1 promotes the expression of TFEB.)	Reduction of huntingtin protein aggregation	22786682
PTM: mTORC-mediated phosphorylation	Alcoholic liver disease	Hepatic steatosis, inflammation, and fibrosis.	Diets of chronic ethanol feeding plus an acute binge	Treatment with Treatment with Torin 1 (a potent mTORC1 inhibitor)	Inhibition of EtOH-induced liver injury and steatosis	29782848
	Sensorineural hearing loss	Progressive degeneration of cochlear nerve fibers and spiral ganglion neurons (SGNs)	A mouse model of SGNs degeneration via injection of kanamycin sulfate	Injection of CCI-779 (an MTOR inhibitor, causing dephosphorylation and nucleus translocation of TFEB by inhibiting MTOR)	Attenuation of SGN and nerve fiber degeneration	30706760
	Alzheimer’s disease	A common neurodegenerative disorder, characterized by the accumulation of protein aggregates including phosphorylated Tau aggregates	Homozygous human P301S Tau transgenic mice	Injection of Celastrol (Celastro activates TFEB via inhibiting mTORC1.)	Reduction of insoluble, phosphorylated Tau aggregates and improvement in memory deficiency	35847498
PTM: MAPK/ERK-mediated phosphorylation	Nonalcoholic fatty liver disease	A metabolic liver disease	Methionine- and choline-deficient diet-fed mice	Treatment with ezetimibe (Ezetimibe induces nuclear TFEB translocation via inhibiting MAPK/ERK-related pathway)	Improvement in hepatic steatosis and fibrosis	28933629
PTM: Akt-mediated phosphorylation	Batten disease	A neurodegenerative disease, characterized by the intralysosomal storage	Cln3^Δex7-8^ mice, an established model of Batten disease	Administration of trehalose (trehalose activates TFEB via inhibiting Akt)	Increasing clearance of proteolipid aggregates, reduction of and extended life of diseased mice	28165011
	Chronic myocardial infarction (MI)	Ischemic injury	Chronic MI for 4 weeks by permanent left anterior descending coronary artery ligation	Treatment with trehalose	Reduction of cardiac hypertrophy, apoptosis and fibrosis	29724354
	Acute kidney injury (AKI)	Sudden renal function decline	Cisplatin-induced AKI mice	Administration of trehalose.	The decreased levels of BUN and serum, and decreased pathological damage	32483422
PTM: p38 MAPK-mediated phosphorylation	Parkinson’s disease	A neurodegenerative disease, characterized by loss of dopaminergic neurons in the substantia nigra and accumulation of α-synuclein	α-synuclein A53T-tg mice model of Parkinson’s disease.	Treatment with p38 MAPK inhibitor SB203580	Reduction of synapsin-1 (a presynaptic protein)	34930303
PTM: SIRT1-mediated deacetylation	Alzheimer’s disease	A neurodegenerative disease, presenting with deposition of β-amyloid (Aβ) in the brain	Incubated fAβ in primary microglia	Treatment with resveratrol, an agonist of SIRT1	Degradation of fAβ	27209302

In contrast, TFEB is hyperactivated and plays a pathogenic role in some cancers, including Birt-Hogg-Dubé (BHD) syndrome [84], TFEB translocation renal cell carcinoma [14, 91], and pancreatic cancer [92]. Reducing the expression has been shown to improve the disease phenotypes of these cancers [69, 92].

BHD syndrome is an autosomal dominant inherited disorder caused by mutations in the Rag C/D activator, *FLCN*, and is characterized by cutaneous fibrofolliculoma, lung and renal cysts, and renal cell carcinoma [84]. Napolitano et al. simulated the kidney phenotype of BHD syndrome in kidney-specific *Flcn* KO mice. Contradictory phenomenon of simultaneous mTORC1 and TFEB activation occurs in *Flcn* KO mice. This is because knockdown of *Flcn* leads to the inactivation of Rag C/D, which in turn leads to aberrant activation of TFEB without phosphorylation by mTORC1 [69]. Constitutive activation of TFEB is a critical determinant of the renal phenotype associated with BHD. TFEB depletion rescued renal pathology and lethality in *Flcn* KO mice [69]. In addition, the *TFEB* gene located on chromosome 6 fuses with *MALAT1*, an α gene of unknown function, resulting in overexpression of TFEB which in turn leads to the development of t (6; 11) translocation renal cell carcinoma [14]. *Tfeb* specifically overexpressed in the distal tubules and collecting ducts of mice, leads to the development of renal cysts and renal cell carcinoma, which are strikingly similar to the renal pathological phenotype in BHD syndrome [69, 93]. Overexpression of TFEB is strongly nephrotoxic [69, 93].

In addition to the kidneys, elevated TFEB expression promotes cancer development in other organs. In pancreatic duct adenocarcinoma (PDA) cell lines, TFEB nuclear localization is increased [43], and TFEB expression is higher in human pancreatic cancer samples than in normal tissue samples [92]. Ji et al. found that TFEB controls glutamine metabolism by promoting the transcription of glutaminase, which meets the biosynthetic needs of cancer cells and supports pancreatic cancer growth. They also found that TFEB knockdown suppressed tumor growth [92].

## Conclusions and perspectives

In this review, we elucidate the regulatory mechanisms of TFEB and the latest research results on the application of these mechanisms to disease treatment. There are still many questions regarding the regulatory mechanisms of TFEB and the use of targeted TFEB as a disease treatment strategy.

It should be noted that the regulatory mechanism of TFEB is not universal in most cells. As mentioned above, phosphorylation of TFEB-S401 by p38-MAPK promoted its nuclear accumulation in undifferentiated THP 1 cells treated with PMA, whereas this was not observed in already differentiated THP 1 cells treated with PMA [46]. In addition, most of the above studies on the regulatory mechanism of TFEB were verified in the normal physiological state of cells in vitro. Whether these regulatory mechanisms exist in animals or pathological states remains to be verified.

Table 2 lists the treatment strategies for TFEB agonists successfully used in several disease models. However, the application of targeted TFEB as a therapeutic strategy in the above disease model has not been evaluated in the long term, and it is unclear whether the long-term application of TFEB agonists is safe and effective. Additionally, the hyperactivation of TFEB may promote tumorigenesis [14, 84]. Therefore, attention should be paid to control the degree of TFEB activation when inducing TFEB activation as a therapeutic strategy for diseases.

A recent study presented the first direct TFEB inhibitor, eltrombopag (EO), a United States Food and Drug Administration-approved drug for the treatment of thrombocytopenia. EO can bind to the basic helix-loop-helix-leucine zipper domain of TFEB and disrupt TFEB-DNA interactions both in vitro and in cellular contexts [94]. Using EO, a new, potent, and safe autophagy inhibitor, the goal of future research will be to develop a combination strategy to benefit a wide range of cancer treatments.

## Data Availability

All data that support the findings of this study are available from the corresponding author upon reasonable request.
